# Spontaneous Gastrojejunal Fistulization after Roux-en-Y Bypass Surgery: A Case Report and Review of Literature

**DOI:** 10.7759/cureus.5035

**Published:** 2019-06-29

**Authors:** Michell J Lopez Luciano, Eric O Then, Christopher J Brana, Andrew Ofosu, Vinaya Gaduputi

**Affiliations:** 1 Internal Medicine, St Barnabas Hospital Health System, Bronx, USA; 2 Internal Medicine, St. Barnabas Hospital Health System, Bronx, USA; 3 Internal Medicine: Gastroenterology, The Brooklyn Hospital Center, Affiliate of the Mount Sinai Hospital, New York, USA

**Keywords:** bariatric surgery, obesity, gastric fistula, endoscopy, leak, roux-en-y gastric bypass, gastrogastric fistula, adverse outcomes following bariatric surgery

## Abstract

Fistula development is an uncommon but well-recognized complication following Roux-en-Y gastric bypass (RYGB). The broad spectrum of clinical presentation represents a challenge at the time of diagnosis. We present the case of a patient who developed gastrojejunal fistulization after gastric bypass surgery.

## Introduction

Obesity is a chronic disease that has shown a significant increase in prevalence over time, and nowadays considered a global epidemic. The most recent epidemiological information offered by the World Health Organization reveals that worldwide obesity prevalence has increased by almost three folds from 1975. In 2016, 39% of the adult population fit into the classification of overweight, and 13% met the criteria for obesity [1].

The adverse effects of obesity for the overall health are not something new for the medical field; in fact, these consequences have been known for more than 2000 years [2]. In 2010, it was estimated that approximately 3.4 million deaths worldwide were related to obesity [3]. Obesity represents the second most common cause of preventable death after tobacco use [4].

The most common comorbidities related to obesity are diabetes, dyslipidemia, hypertension, which are all main risk factors for the development of cardiovascular diseases, the number one cause of death worldwide [5]. Other remarkable diseases related to obesity are osteoarthritis, gastroesophageal reflux disease (GERD), multiple types of cancer and, not less important, the significant psychological effect that this has on the affected patients.

Given all the complications mentioned above, and the fact that most of the obesity-related comorbidities can be resolved or significantly controlled by adequate weight loss, many efforts have been made over the years to treat obesity and achieve perdurable effects. From all the treatments available, bariatric surgery is considered the most effective long-term treatment for morbid obesity, the reason why this procedure has become so popular over the years. According to the American Society for Metabolic and Bariatric Surgery, bariatric surgery is recommended for patients with a body mass index equal or more than 40 kg/m^2^ or 35 kg/m^2^ with associated comorbidities and that have not had an adequate response to other medical intervention. Epidemiologic data offered by the American Society for Metabolic and Bariatric Surgery showed that in 2015 in the USA approximately 196,000 bariatric surgeries were performed - 17,000 more cases compared to 2013, representing a 9.5% increase in the number of cases done. Gastric sleeve surgery represents more than half of the cases, followed by Roux-en-Y gastric bypass [6, 7].

As expected, any increase in the frequency of a procedure usually unveils a significant number of related complications. Most of the complications of bariatric surgery are usually observed in the postoperative period. Gastric fistulas are the most common complication observed after gastric sleeve surgery. A meta-analysis of almost 10,000 sleeve gastrectomies conducted by Parikh et al. revealed that 2.2% of these procedures were posteriorly complicated by fistula development. In regards to Roux-en-Y gastric bypass (RYGB), anastomotic leaks are their most common complications with an incidence of 0-8% [8,9].

Gastric leaks are interruptions in the surgical anastomosis created in bariatric surgery, resulting in leakage of the luminal content. Fistulas are defined as an abnormal communication between two hollow viscera or the skin. These terms are also used interchangeably very frequently [10]. More than 50% of gastric fistulas have a malignant etiology; the rest usually develop as a consequence of surgical procedures, previous stent placement, tuberculosis, Crohn's Disease, iatrogenic, trauma and acquired immunodeficiency syndrome (AIDS) [10]. Clinical presentation for gastric fistulas is usually very non-specific; this can range from entirely asymptomatic patients to those presenting with abdominal pain, dyspepsia, nausea, and vomiting [11].

## Case presentation

**Figure 1:**
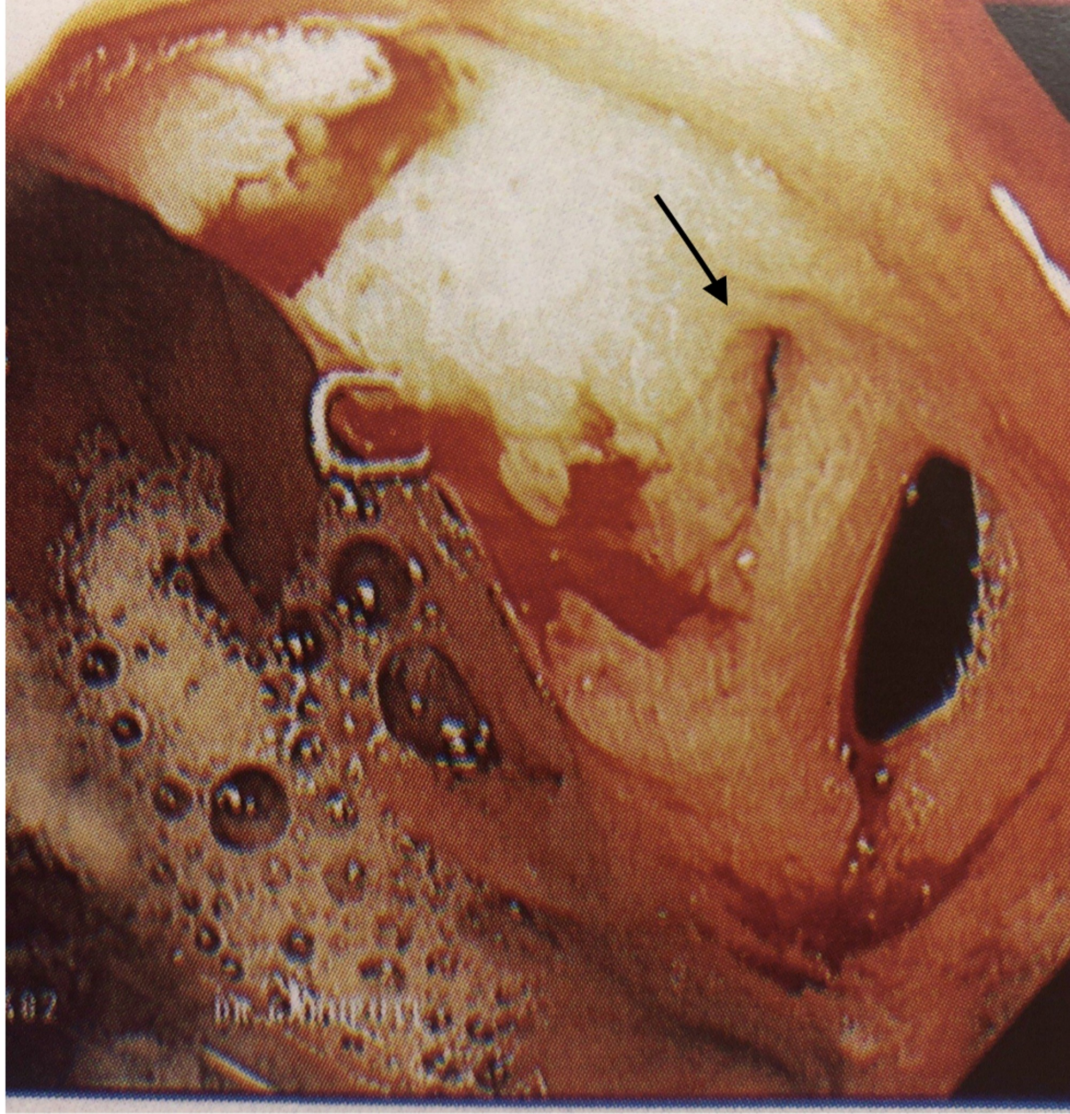
Fistulous opening in the distal gastric pouch.

A 51-year-old woman with a history of gastric bypass in 2007 presented to the clinic with worsening epigastric abdominal pain associated with nausea, and unintentional weight loss of 8 pounds over two-week period. She denied vomiting, hematemesis, melena or hematochezia. Physical examination was unremarkable. Laboratory investigation revealed a normal complete blood count and basic metabolic profile. An esophagogastroduodenoscopy (EGD) showed a small gastric pouch consistent with prior gastric bypass, erythematous mucosa at the gastroenteric anastomosis and a fistulous opening in the distal gastric pouch near the anastomosis (Figure 1). The opening into the jejunal loop was narrowed precluding intubation with an upper endoscope. Biopsies of the anastomotic site exhibited mild chronic inactive gastritis with a negative Helicobacter pylori biopsy. The patient was managed with proton pump inhibitors (PPIs) daily for eight weeks. On follow-up, she reported a complete resolution of her symptoms. A follow-up EGD showed healing of the fistulous tract.

## Discussion

Several risk factors have been associated with a higher risk of fistulas development after bariatric surgery; the most commonly described are body mass index, hypertension, diabetes mellitus, age older than 55, obstructive sleep apnea (OSA), male sex and prior surgical procedures [12]. A review of 78 cases of gastrojejunocolic fistulas performed by Ishi et al., revealed that these are predominantly more common in males and the mean age of diagnosis is usually 45 years [6]. The most common symptoms reported are diarrhea, weight loss, and fecal eructation [7]. Other common findings are also anemia, leukocytosis, electrolyte imbalance, and hypoalbuminemia [8].

The pathophysiology underlying the development of gastric fistulas is most of the times multifactorial, but with the common characteristic in all the situations, that intraluminal pressure overcomes tissue resistance along the surgical anastomosis or staple line, consequently leading to fistula formation [6].

The etiology behind leaks and fistulas development is usually based on the timing after surgery. Early leaks development (less than one week after surgery) is usually related to technical errors like stapler malfunction or high anastomotic tension. On the other hand, ischemia at the anastomosis site or the staple line is the most common etiology on later fistula development [3,13].

Multiple etiologies for gastric fistulas development after RYGB have been described over the years, the most common one reported is poor surgical technique, specifically an inadequate division of the proximal stomach. Other important factors related to fistula development are suture migration, gastric band related, staple line leak, and idiopathic etiology [11].

The most common types of fistulas by location are gastrogastric and gastrocutaneous fistulas. Gastrogastric fistulas are usually related to RYGB, in which there is a communication between the gastric pouch and the gastric remnant [3].

As in most clinical entities, a high clinical suspicion is necessary to diagnose gastric fistulas correctly. The clinical presentation can range from a completely asymptomatic patient to a myriad of non-specific gastrointestinal symptoms, most frequently abdominal pain, nausea, vomiting, belching, pyrosis, recurrence of diabetes mellitus, excessive weight loss or even weight gain and symptoms of peptic ulcer disease (PUD). The main issue with this clinical entity is the non-specificity of its symptoms and the fact that many patients present many of these symptoms in the absence of gastric fistulas after bariatric surgery [11].

The optimal diagnostic studies for gastric fistulas are upper endoscopy and barium contrast studies [11]. Upper endoscopy will commonly demonstrate a granulated fistula tract, that due to its small size will not permit adequate passage of the gastroscope, as seen in our patient. Other cross-sectional imaging studies are useful for ruling out any other differential diagnoses rather than diagnosing gastric fistulas [14]. Ultimately, the critical point in the correct diagnosis of gastric fistulas is, more than anything, a high index of suspicion.

There are three different approaches for the management of fistulas; these are conservative management, endoscopic treatment, and surgical intervention. The choice of the type of treatment will depend on many factors, including the size of the defect, location, and accessibility of the fistula, hemodynamic stability of the patient, timing of diagnosis and availability of resources. Until recently, most of the fistulas were managed surgically; now, the preferred approach is less invasive methods like endoscopic therapy and if suitable, conservative management [10].

The advent of effective anti-ulcer medications, especially H2 blockers and proton pump inhibitors and the establishment of adequate Helicobacter pylori (H. pylori) eradication therapy, has significantly decreased the need for surgery in the management of peptic ulcer disease. Smoking, nonsteroidal anti-inflammatory drugs (NSAIDs) use, alcohol intake, and chronic anticoagulation can also predispose persons with prior gastric surgery to postoperative marginal ulceration and consequent development of gastric fistulas [3,5]. This kind of approach should be attempted only in hemodynamically stable patients, which usually represent approximately 40% of the cases [15]. The primary strategy is to decrease acid secretion, which will allow gastrojejunal anastomosis ulcer to heal, or prevent its development. It has also been proved to promote spontaneous resolution of some types of gastric fistulas without any surgical or endoscopic intervention, as we could appreciate in the management of our patient. Another important consideration is that patients should always be screened and treated, when needed, for H. pylori [11].

Several endoscopic techniques have been developed over the years, including endoscopic suture, placement of metal clips, fibrin glue, or stent placement. Best results with the endoscopic approach are usually seen in less than 10 mm fistulas. Endoclips have the advantage of being well known to endoscopists and less expensive compared to other techniques but have the limitation that, due to their jaw opening, most of them are usually limited for fistulas measuring less than 1 cm [11]. Another endoscopic technique that has shown a high success rate over the years is stent placement. The way these stents work is by decreasing intraluminal pressure, which is the primary mechanism of fistula development. A meta-analysis performed by Okazaki et al. showed a success rate of approximately 73% in patients presenting fistulas after gastric sleeve and RYGB. Some of the complications associated with stent placement are stent migration, bleeding, and perforation. Stent migration represents by far the most significant challenge related to stent placement, observed in approximately 28-30% of the cases. The techniques applied to avoid this complication are a fixation with clips, endoscopic sutures, and the development of longer stents [6]. Despite these measures, migration still represents a challenge in the management of fistulas, reason why it is usually recommended to remove these stents after 6-8 weeks to permit correct closure and avoid excessive tissue hyperplasia.

Large fistulas and failed endoscopic treatment are the most common indications for surgical management. Gastric fistulas have been classified based on their appearance and location in type 1 and type 2; the surgical approach is usually determined based on type. Type 1 fistulas are located more than 1 cm above the anastomosis while type 2 is less than 1 cm from the anastomosis. For type 1 fistulas it is recommended to excise the fistula keeping the gastrojejunal anastomosis. On the other hand, on type 2 fistulas, there is usually the presence of marginal ulcers; for this, the recommendation is a complete revision of anastomosis and excision of the fistula [16]. The most common surgical approach is radical en bloc removal of the fistula and reconstruction of the physiologic gastrojejunal passage in addition to correct parenteral and enteral support treatment [9].

## Conclusions

Gastric fistulas are a significant complication of gastric bypass surgery that can be seen up to 20 years after the procedure. This entity should always be considered in the differential diagnosis of all postoperative gastric bypass patients who present with abdominal pain. Early diagnosed gastric fistulas can be successfully treated with PPI therapy, reducing diagnostic and therapeutic cost. This case further validates that surgical intervention is not always required in gastroenteric fistulas and that medical therapy with appropriate follow-up can be used to manage such complications.
